# Animal sources for zoonotic transmission of psittacosis: a systematic review

**DOI:** 10.1186/s12879-020-4918-y

**Published:** 2020-03-04

**Authors:** Lenny Hogerwerf, Inge Roof, Marianne J. K. de Jong, Frederika Dijkstra, Wim van der Hoek

**Affiliations:** 1 Centre for Infectious Disease Control, National Institute for Public Health and the Environment, Bilthoven, the Netherlands; 2 Faculty of Veterinary Medicine, Utrecht University, Utrecht, the Netherlands

**Keywords:** Psittacosis, Chlamydia psittaci, Zoonosis, Animal sources, Strength of evidence

## Abstract

**Background:**

Human psittacosis, caused by *Chlamydia (C.) psittaci*, is likely underdiagnosed and underreported, since tests for *C. psittaci* are often not included in routine microbiological diagnostics. Source tracing traditionally focuses on psittacine pet birds, but recently other animal species have been gaining more attention as possible sources for human psittacosis. This review aims to provide an overview of all suspected animal sources of human psittacosis cases reported in the international literature. In addition, for each animal species the strength of evidence for zoonotic transmission was estimated.

**Methods:**

A systematic literature search was conducted using four databases (Pubmed, Embase, Scopus and Proquest). Articles were included when there was mention of at least one human case of psittacosis and a possible animal source. Investigators independently extracted data from the included articles and estimated strength of evidence for zoonotic transmission, based on a self-developed scoring system taking into account number of human cases, epidemiological evidence and laboratory test results in human, animals, and the environment.

**Results:**

Eighty articles were included, which provided information on 136 different situations of possible zoonotic transmission. The maximum score for zoonotic transmission was highest for turkeys, followed by ducks, owls, and the category ‘other poultry’. Articles reporting about zoonotic transmission from unspecified birds, psittaciformes and columbiformes provided a relatively low strength of evidence. A genotypical match between human and animal samples was reported twenty-eight times, including transmission from chickens, turkeys, guinea fowl, peafowl, pigeons, ducks, geese, songbirds, parrot-like birds and owls.

**Conclusions:**

Strong evidence exists for zoonotic transmission from turkeys, chickens and ducks, in addition to the more traditionally reported parrot-like animal sources. Based on our scoring system, the evidence was generally stronger for poultry than for parrot-like birds. Psittaciformes should not be disregarded as an important source of human psittacosis, still clinicians and public health officials should include poultry and birds species other than parrots in medical history and source tracing.

## Background

Psittacosis is a zoonosis caused by the bacterium *Chlamydia (C.) psittaci*. Transmission occurs mainly by inhalation of the agent, which is excreted by birds in feces and in droplets from the respiratory tract [1, 2]. In the 1930s, major outbreaks of psittacosis occurred worldwide, caused by trade of parrots and other tropical birds. These outbreaks are even referred to as ‘the psittacosis pandemic’ [3–5]. In 1985, an outbreak of psittacosis affected employees at a duck processing plant in the United Kingdom [6]. Nowadays psittacosis cases are usually limited to local outbreaks, small clusters or isolated cases [7–17]. Community-acquired pneumonia (CAP) is the most important presentation of human psittacosis, but microbiological testing for psittacosis in a pneumonia patient is often not incorporated in routine diagnostics. Due to the non-specific symptoms and the fact that often only severely ill patients are being tested for *C. psittaci*, the disease is likely underdiagnosed and underreported [18–22]. A recent review and meta-analysis of CAP etiological studies estimated that in 1.03% (95% CI: 0.79–1.30) of all CAP cases from the included studies combined, *C. psittaci* was the causative pathogen, with a range between studies from 0 to 6.7% [21]. Based on this result, an estimated 4.4% (95% CI: 1.6–8.2%) of symptomatic cases were notified in the Netherlands over the period 2012–2014 [20]. Human psittacosis is mostly linked to parrots or ornamental birds as source of infection, however, recently other bird and animal species have been gaining more attention as potential sources of human psittacosis, such as poultry species, pigeons and even mammals [23–26]. To inform clinicians, public health officials and people at risk of exposure to potentially infected animals, we reviewed animal sources that have been associated with human psittacosis in the recent international literature and provided strength of evidence for zoonotic transmission for each of the animal categories.

## Methods

### Search strategy

A literature search of studies describing human cases of psittacosis with an associated animal source was conducted. The databases Pubmed, Embase, Scopus and Proquest (CAB Abstracts and BIOSIS Previews) were searched using the following terms and synonyms hereof: psittacosis, *Chlamydia* or *Chlamydophila psittaci*, psittaci, ornithosis, human, patient and zoonosis. Studies were included from 1 January 2000 to 27 June 2018, because of the major adjustments in taxonomy and nomenclature from the year 1999 [27]. Languages were restricted to Dutch, German, Spanish, French, Portuguese and English. No limitations were applied regarding the study design. The search results from all databases were merged into one EndNote X8 file and removal of duplicates was performed using EndNote and by hand.

### In- and exclusion criteria

Titles and abstracts were screened by two investigators (IR and MdJ) and records were included when there was mention of at least one human case of psittacosis and a possible animal source. Records without abstract were included based on relevance of the title. Reports mentioning only animal sources without human cases were excluded. Full-text assessment was performed by two investigators (IR and MdJ) and uncertainties about article inclusion were discussed with other authors (FD, LH or WvdH). Exclusion criteria during full-text assessment were: no laboratory confirmed human cases, no animal source, no specification of animal exposure (e.g. animal, pet, zoo, veterinarian), review articles, guidelines, articles presenting unoriginal data (e.g. mentioning identical cases and identical associated animal sources as previously reported without additional evidence), human psittacosis due to *Chlamydia* species other than *C. psittaci* and language other than Dutch, Spanish, French, Portuguese, English or German. Reference lists of included full-text articles were screened by hand for additional titles.

### Data extraction

Four investigators (IR, LH, MdJ and WvdH) independently extracted the following data from the included articles: year and country of human cases, animal species, number of human cases, contact of human case with sick animal, type of diagnostics used in humans/animals with associated results, genotyping results in humans/animals, environmental investigation and epidemiological evidence. Any disagreement was resolved through discussion and consensus. During data extraction the rationale and definitions of the original authors were followed. The total number of confirmed human cases was defined by adding the number of probable cases (according to the original author’s definition of a probable case) and the number of confirmed human cases with a positive laboratory result. We did not specify criteria for laboratory results because there is a wide variety in criteria for confirmation of a human case in the literature. Analysis of animal faecal samples was considered as environmental investigation. Epidemiological evidence was confirmed when a study demonstrated an increased risk of human psittacosis by the animal species involved. When studies reported multiple situations of zoonotic transmission with different animal sources or when cases had multiple associated animal exposures, the animal species were entered in separate lines under the same study. In addition, studies reporting multiple unrelated cases were also entered separately. Bird species were categorized according to their order in the bird taxonomy (e.g. psittaciformes, passeriformes, columbiformes etc.). Poultry species were separated into the categories chicken, duck, turkey and other poultry. Situations reporting on bird or poultry species without further specification were included under the category ‘unspecified birds’ or ‘unspecified poultry’ respectively.

### Calculation strength of evidence

Strength of evidence for zoonotic transmission was calculated based on a scoring system using the following factors (weight between brackets): number of confirmed human cases above the overall median number calculated across the included studies (2); positive antibody test in humans (1) or animals (1); detection of antigen in humans (2) or animals (2); genotyping results in humans (2) or animals (2); contact with sick animal (2); environmental sample positive for *C. psittaci* antigen (2); genotyping of environmental sample (2); epidemiological evidence (4); genotypical match between human and animal species category (8), genotypical mismatch between human and animal species category (set final score to 0). The strength of evidence score was calculated per animal species per study or per animal species for each separate case when a study reported multiple unrelated cases.

## Results

### Inclusion of articles

**Fig. 1:**
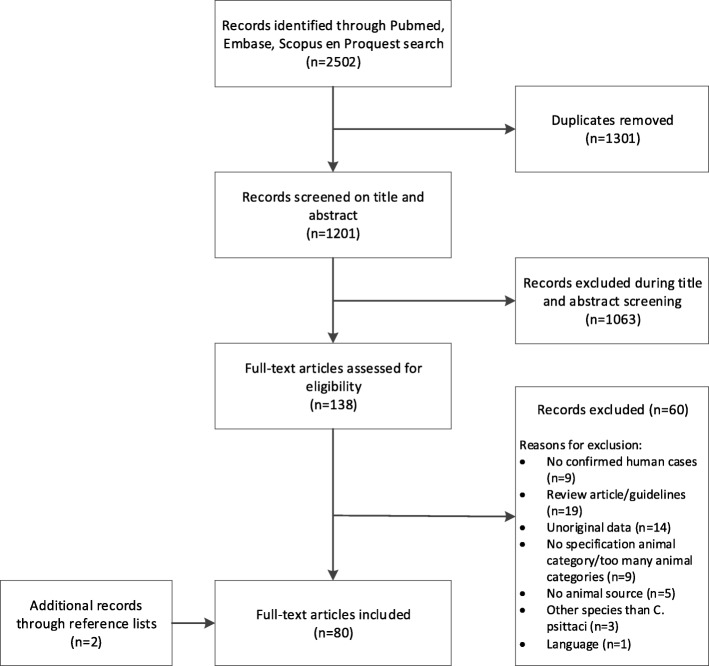
Flow chart of literature search and selection of articles

The search strategy yielded 2502 articles from four databases, of which 1201 were unique and screened for eligibility on title and abstract. Of all 138 articles included in the full-text screening, the full-text could be retrieved. Two additional records were found by screening the reference lists of included full-text articles. In total, 80 articles met the criteria for final inclusion in this review (Fig. 1). No quality assessment of study design was performed, because the majority of studies were case reports.

### Characteristics of included articles

The selected 80 articles described 136 associations of human psittacosis cases with an animal source (Table 1). The largest number of articles originated from Belgium (23%), the Netherlands (10%) and France (10%). Thirteen studies were cross-sectional or seroprevalence studies, investigating the prevalence of *C. psittaci* infection in high-risk groups and animals, for example occupationally exposed individuals or people living in areas with high animal or bird density [25, 28, 29, 34, 44, 45, 63, 68, 73, 76, 77, 88, 90]. Fifteen articles reported on outbreaks or prevalence of human psittacosis related to (mixed) poultry processing plants, farms or slaughterhouses (e.g. turkey, chicken and duck) [8, 28, 30, 45, 46, 68, 73–75, 77, 88, 91, 92, 95, 96]. Other included articles described psittacosis cases in relation to a bird show or bird park [7, 13, 31], veterinary clinic or teaching hospital [33, 37, 65, 89], and a pet shop [10, 42, 50, 58, 94]. Psittaciformes were mentioned as animal source in 40 of the 136 (29%) included associations, followed by columbiformes (*n* = 21, 15%) and chickens (*n* = 15, 11%). For eleven and four associations respectively, the bird or poultry species were not specified. Also mammalian species (e.g. horse, cattle, pig, goat, sheep, fox and dog) were considered as psittacosis source in eight instances. In 39 of the 136 (29%) associations, there had been contact with a sick animal. In the large majority (58%), however, contact with a sick animal was considered unknown. The characteristics and relevant extracted data of all included studies are listed in Additional file 1.

**Table 1:** Included studies describing human psittacosis cases and associated animal sources with strength of evidence score

Reference, year	Animal species	Year of notification	Country	Diagnostics human	No. confirmed human cases	Diagnostics animal and/or environment	Strength of evidence
Abadia et al., 2006 [28]	ducks	2000	France	serology	71^b^	no	8
	chickens	2000	France	serology	71^b^	no	4
	turkeys	2000	France	serology	71^b^	no	4
Arenas-Valls et al., 2017 [17]	psittaciformes	unknown	Spain	PCR & serology	4	no	8
Arraiz et al., 2012 [29]	columbiformes	unknown	Venezuela	PCR	8	PCR	19
Belchior et al., 2010 [30]	ducks	2009	France	PCR & serology	4	no	12
Belchior et al., 2011 [31]	unspecified birds	2008	France	PCR & serology	4	PCR	8
Berk et al., 2008 [7]	passeriformes	2007	The Netherlands	PCR & serology	12	antigen detection	8
Bourne et al., 2003 [32]	psittaciformes	unknown	United Kingdom	serology	1	no	2
Branley et al., 2008 [33]	psittaciformes	unknown	Australia	PCR, culture & serology	3	PCR & culture	12
Branley et al., 2014 [34]	unspecified birds	2003–2009	Australia	PCR, culture & serology	48	no	6
Cadario et al., 2017^a^ [35]	chickens	2010	Argentina	PCR	1	no	5
	psittaciformes	2011	Argentina	PCR	1	PCR	17
	psittaciformes	2013	Argentina	PCR	1	PCR	17
	columbiformes	2013	Argentina	PCR	1	no	5
	psittaciformes	2014	Argentina	PCR	1	no	5
Carlier et al., 2014 [36]	ducks	2011	France	PCR & serology	1	PCR	10
Chan et al., 2017 [37]	horse	2014	Australia	serology	3	PCR & serology	15
Chau et al., 2015^a^ [38]	psittaciformes	2014	Hong Kong	PCR & serology	1	no	4
	chickens	2014	Hong Kong	PCR & serology	1	no	4
	geese	2014	Hong Kong	PCR & serology	1^b^	no	4
	chickens	2014	Hong Kong	PCR & serology	1^b^	no	4
Cheng et al., 2013 [39]	psittaciformes	2011	Taiwan	serology	1	no	4
Ciftci et al., 2008 [40]	psittaciformes	unknown	Turkey	serology	2	no	2
Clarence et al., 2016 [41]	columbiformes	unknown	United Kingdom	PCR	1	no	5
De Boeck et al., 2016 [42]	psittaciformes	2013	Belgium	PCR, culture & serology	3	PCR	22
De Schrijver et al., 2016 [43]	psittaciformes	2014	Belgium	serology	4	PCR	10
Dickx et al., 2010 [44]	columbiformes	2008	Belgium	PCR & culture	4	PCR & culture	19
Dickx et al., 2010 [45]	chickens	2007	Belgium	PCR, culture & serology	43	PCR & culture	14
	turkeys	2007	Belgium	PCR, culture & serology	33	PCR & culture	22
Dickx et al., 2011 [46]	chickens	2010	Belgium	PCR & culture	4^b^	PCR & culture	19
	guinea fowl	2010	Belgium	PCR & culture	4^b^	PCR & culture	19
	turkeys	2010	Belgium	PCR & culture	4^b^	PCR & culture	19
Dovc et al., 2005 [47]	psittaciformes	1997	Slovenia	serology	1	serology	3
Dovc et al., 2007 [48]	psittaciformes	unknown	Slovenia	serology	1	PCR & serology	6
Elliot et al., 2001 [49]	unspecified birds	unknown	Australia	serology	1	no	2
Espinosa de los Monteros et al., 2005 [50]	psittaciformes	2003	Spain	PCR & serology	3	PCR & serology	9
Fenga et al., 2007 [25]	cattle	2005	Italy	serology	28^b^	no	8
	pigs	2005	Italy	serology	28^b^	no	8
	goats	2005	Italy	serology	28^b^	no	8
	sheep	2005	Italy	serology	28^b^	no	8
Ferreira et al., 2015 [51]	psittaciformes	unknown	Brazil	serology	3	PCR	8
Ferreira et al., 2017 [52]	psittaciformes	unknown	Brazil	serology	1	PCR	6
Ferreri et al., 2007 [53]	passeriformes	2000	Italy	PCR & immunohistochemistry	1	PCR & immunohistochemistry	17
Fraeyman, 2010^a^ [54]	columbiformes	unknown	Belgium	PCR & serology	1	no	3
	columbiformes	unknown	Belgium	PCR & serology	1	no	3
	columbiformes	unknown	Belgium	PCR & serology	1	unknown	3
Frutos et al., 2012 [55]	psittaciformes	2010–2011	Argentina	PCR	6^b^	no	7
	chickens	2010–2011	Argentina	PCR	1^b^	no	5
	columbiformes	2010–2011	Argentina	PCR	1^b^	no	5
	passeriformes	2010–2011	Argentina	PCR	2^b^	no	5
Gacouin et al., 2012 [56]	chickens	1993–2011	France	PCR or serology	8^b^	no	3
	ducks	1993–2011	France	PCR or serology	8^b^	no	3
	psittaciformes	1993–2011	France	PCR or serology	2	no	1
	columbiformes	1993–2011	France	PCR or serology	2	no	1
Gaede et al., 2008 [8]	chickens	2005	Germany	PCR & serology	7^b^	PCR	22
	ducks	2005	Germany	PCR & serology	7^b^	PCR	22
	geese	2005	Germany	PCR & serology	7^b^	PCR	22
Garbim et al., 2017 [57]	psittaciformes	unknown	Brazil	serology	1	no	2
García-Ordóñez et al., 2011 [58]	psittaciformes	2009	Spain	serology	5	PCR	6
Geens et al., 2005 [59]	turkeys	unknown	Belgium	PCR	1	PCR	19
Gelfand et al., 2013 [60]	psittaciformes	unknown	United States of America	serology & immunohistochemistry	2	PCR & immunohistochemistry	6
Haas et al., 2006 [9]	columbiformes	unknown	The Netherlands	serology	1	PCR	2
Haas et al., 2007 [61]	ducks	2007	Germany	PCR	1	no	5
Harkinezhad et al., 2007 [62]	psittaciformes	unknown	Belgium	PCR, culture & serology	3	PCR & culture	22
Harkinezhad et al., 2009 [63]	psittaciformes	2002–2003	Belgium	PCR & serology	8	no	10
	columbiformes	2002–2003	Belgium	PCR & serology	8	no	10
	passeriformes	2002–2003	Belgium	PCR & serology	12	no	10
	turkeys	2002–2003	Belgium	PCR & serology	1	no	4
Heddema et al., 2003 [64]	columbiformes	unknown	The Netherlands	PCR & serology	1	PCR	4
Heddema et al., 2006 [65]	psittaciformes	2005	The Netherlands	PCR & serology	10^b^	PCR	20
	columbiformes	2005	The Netherlands	PCR & serology	10^b^	PCR	0
Heddema et al., 2015 [66]	psittaciformes	2008–2013	The Netherlands	PCR	8^b^	no	7
	passeriformes	2008–2013	The Netherlands	PCR	5^b^	no	7
	columbiformes	2008–2013	The Netherlands	PCR	10^b^	no	7
	ducks	2008–2013	The Netherlands	PCR	1^b^	no	5
	pheasants	2008–2013	The Netherlands	PCR	1^b^	no	5
	unspecified poultry	2008–2013	The Netherlands	PCR	5^b^	no	7
	unspecified birds	2008–2013	The Netherlands	PCR	9^b^	no	7
Henrion et al., 2002 [67]	psittaciformes	2001	Belgium	serology	1	no	2
Hulin et al., 2015 [68]	ducks	2013	France	PCR & serology	10	PCR	8
	chickens	2013	France	PCR & serology	7^b^	PCR	4
	turkeys	2013	France	PCR & serology	7^b^	PCR	4
	guinea fowl	2013	France	PCR & serology	7^b^	PCR	4
	unspecified poultry	2013	France	PCR & serology	7^b^	PCR	8
Ionescu et al., 2016 [69]	psittaciformes	unknown	United Kingdom	PCR & serology	1^b^	no	6
	passeriformes	unknown	United Kingdom	PCR & serology	1^b^	no	6
	columbiformes	unknown	United Kingdom	PCR & serology	1^b^	no	6
	chickens	unknown	United Kingdom	PCR & serology	1^b^	no	6
Ito et al., 2002 [10]	psittaciformes	unknown	Japan	serology	1	no	2
Jiménez-Cordero et al., 2015 [70]	columbiformes	unknown	Spain	serology	1	no	2
Kaibu et al., 2006 [11]	psittaciformes	2005	Japan	PCR & serology	2	PCR & culture	6
Kalmar et al., 2014 [71]	columbiformes	unknown	Belgium	PCR & culture	3^b^	PCR & culture	21
	passeriformes	unknown	Belgium	PCR & culture	3^b^	PCR & culture	19
	strigiformes	unknown	Belgium	PCR & culture	3^b^	PCR & culture	21
Kampinga et al., 2000 [72]	sheep	unknown	The Netherlands	PCR & serology	1	no	6
Kovacova et al., 2007 [12]	psittaciformes	2005	Slovakia	PCR & serology	1	serology	5
Lagae et al., 2014 [73]	chickens	2012	Belgium	PCR & culture	29	PCR & culture	21
Laroucau et al., 2009 [74]	ducks	2006	France	PCR & serology	5	PCR & culture	20
Laroucau et al., 2015 [75]	chickens	2013	France	PCR & serology	5^b^	PCR & culture	20
	ducks	2013	France	PCR & serology	5^b^	PCR	20
Ling et al., 2015 [76]	columbiformes	2008–2010	China	Antigen detection & serology	19	Antigen detection & serology	21
Lugert et al., 2017 [77]	ducks	2010	Germany	serology	5	no	8
Mair-Jenkins et al., 2018 [78]	columbiformes	2015	United Kingdom	PCR & serology	4	no	8
Matsui et al., 2008 [13]	unspecified birds	2001–2002	Japan	serology	8	PCR	12
Maza et al., 2016 [79]	psittaciformes	2014	Argentina	PCR	2	PCR & immunohistochemistry	5
Pandeli et al., 2006 [80]	psittaciformes	unknown	Australia	PCR	1^b^	no	5
	fox	unknown	Australia	PCR	1^b^	no	5
Petrovay et al., 2008 [81]	unspecified poultry	2005	Hungary	PCR & serology	1	no	4
	unspecified poultry	2007	Hungary	PCR & serology	1	no	4
Rehn et al., 2013 [82]	unspecified birds	2013	Sweden	PCR	15^b^	no	11
	psittaciformes	2013	Sweden	PCR	1^b^	PCR	5
	chickens	2013	Sweden	PCR	1^b^	PCR	5
Sciacca et al., 2009 [83]	psittaciformes	2009	Belgium	serology	1	no	2
Senn et al., 2008 [84]	psittaciformes	2007	Switzerland	serology	1	PCR	4
Speelberg et al., 2014^a^ [85]	musophagiformes	unknown	The Netherlands	PCR & serology	1	no	7
	columbiformes	unknown	The Netherlands	PCR & serology	1	PCR	7
	chickens	unknown	The Netherlands	PCR & serology	1	PCR	5
Spoorenberg et al., 2016^a^ [86]	unspecified birds	2007–2010	The Netherlands	PCR & serology	1	no	8
	psittaciformes	2007–2010	The Netherlands	PCR & serology	1	no	6
	unspecified birds	2007–2010	The Netherlands	PCR & serology	1	no	4
	unspecified birds	2007–2010	The Netherlands	PCR & serology	1	no	6
	columbiformes	2007–2010	The Netherlands	PCR & serology	1	no	3
	unspecified birds	2007–2010	The Netherlands	PCR & serology	1	no	3
Sprague et al., 2009 [87]	dogs	2006–2007	Germany	culture & serology	2	PCR & culture	10
Telfer et al., 2005 [15]	unspecified birds	2002	Australia	serology	28	no	8
Tiong et al., 2007 [88]	ducks	2003–2004	Australia	serology	12	culture & serology	11
Van Droogenbroeck et al., 2009 [89]	turkeys	unknown	Belgium	PCR & culture	1	PCR & culture	19
Vande Weygaerde et al., 2018 [16]	psittaciformes	unknown	Belgium	PCR & serology	1	PCR	17
Vanrompay et al., 2007 [90]	psittaciformes	unknown	Belgium	PCR & culture	6	PCR & culture	9
Verminnen et al., 2008 [91]	turkeys	unknown	Belgium	PCR, culture & serology	3	PCR, culture & serology	25
Vorimore et al., 2015 [92]	ducks	2009	Belgium	serology	4	PCR	8
Walter et al., 2014 [93]	psittaciformes	unknown	United Kingdom	serology	1	no	2
Widgren et al., 2009 [94]	psittaciformes	2008	Denmark	serology	4	unknown	10
Williams et al., 2013 [95]	ducks	2008	United Kingdom	culture & serology	9	no	10
Yang et al., 2011 [96]	peacock	2009	China	PCR & serology	4	PCR & serology	23

^a^ Studies reporting on multiple unrelated case studies

^b^ Cases with multiple associated animal sources

### Diagnostics in human and animal

Most studies used polymerase-chain-reaction (PCR), serology or a combination of PCR and serology for human diagnostics (Table 1). In around half (71/136) of the human-animal associations, no diagnostics regarding animals and/or the environment were performed. When animal diagnostics were carried out, mostly PCR was used. In 55% of the situations when PCR was applied for human diagnostics, genotyping of the *C. psittaci* strain was also performed.

### Strength of evidence across animal categories

**Fig. 2:**
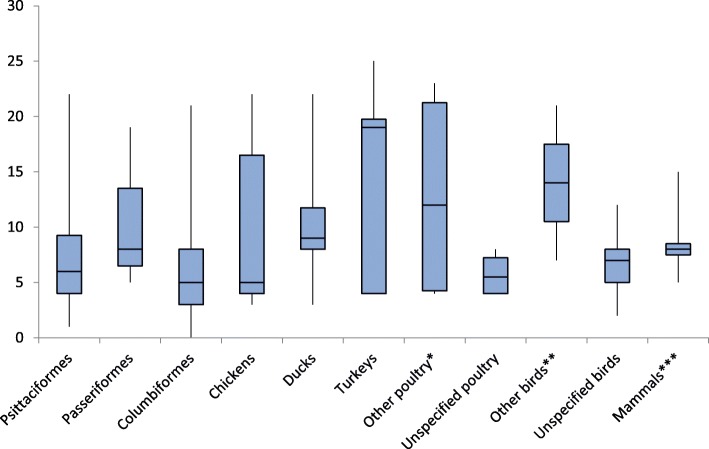
Distribution of strength of evidence across different animal categories. The lower whisker includes the first 25% of scores (first quartile); the box includes the second and third quartile separated by the median and the top whisker represents the last quartile of the scores. Outliers were not excluded because results were not within a normal distribution and outliers are of particular interest for this review. Therefore, the upper end of the top whisker represents the maximum score and the lower end of the bottom whisker represents the minimum score. * geese, guinea fowl, pheasants, peacock. ** strigiformes, musophagiformes. *** horse, cattle, pig, goat, sheep, fox, dog

Figure 2 presents the distribution of strength of evidence for zoonotic transmission by animal category in boxplots. High maximum scores for strength of evidence were obtained for turkeys (25), chickens (22), ducks (22), psittaciformes (22), columbiformes (21) and passeriformes (19). The category ‘other poultry’, including geese, guinea fowl, pheasant and peacock, had also a high maximum score of 23. ‘Unspecified poultry’ and ‘unspecified birds’ only had a maximum score of 8 and 12 respectively. Median scores for strength of evidence were highest for turkeys (19). The single description of zoonotic transmission from strigiformes and peacock had a relatively high strength of evidence score of 21 and 23 respectively [71, 96]. For both geese and guinea fowls, two descriptions of animal-human transmission were found, with one description scoring low (4) [38, 68] and the other scoring high (geese (22) [8], guinea fowl (19) [46]). The single association with dogs had a score of 10 and two single reports from a fox and pheasant scored relatively low with 5 points each [66, 80, 87]. In the scoring system used in this article, the factor ‘genotypical match’ was given the highest weight (i.e. 8 points). Additional file 2 provides an interactive version of the strength of evidence tool, allowing the reader to replace the default scores by user-defined scores. A genotypical match between the human and animal or environmental samples was found for the animal categories chicken, columbiformes, ducks, geese, guinea fowl, passeriformes, peacock, psittaciformes, strigiformes and turkeys (Table 2). Ferreri et al. concluded that patient and animal were infected by the same *C. psittaci* strain, however, the genotype was not specified [53]. The association with columbiformes from Heddema et al. (2006) had an strength of evidence of zero, because of a genotypical mismatch between the human and animal samples [65].

**Table 2:** Overview of studies demonstrating a genotypical match between human and animal or environmental samples

Animal category	Reference	Genotype human	Genotype animal	Genotype environment
Chickens	Dickx et al., 2011 [46]	A, C & D	–	D
	Gaede et al., 2008 [8]	A	A & E/B	–
	Lagae et al., 2014 [73]	A, C & D	A & D	–
	Laroucau et al., 2015 [75]	E/B	E/B	–
Columbiformes	Arraiz et al., 2012 [29]	B	B	–
	Dickx et al., 2010 [44]	D	–	D
	Kalmar et al., 2014 [71]	B	B	–
	Ling et al., 2015 [76]	B	B	–
Ducks	Gaede et al., 2008 [8]	A	A & E/B	–
	Laroucau et al., 2009 [74]	E/B	E/B	–
	Laroucau et al., 2015 [75]	E/B	E/B	–
Geese	Gaede et al., 2008 [8]	A	A & E/B	–
Guinea fowl	Dickx et al., 2011 [46]	A, C & D	–	A & C
Passeriformes	Ferreri et al., 2007 [53]	unknown	unknown	–
	Kalmar et al., 2014 [71]	B	A & B	–
Peacock	Yang et al., 2011 [96]	B	B	–
Psittaciformes	Cadario et al., 2017 [35]	A	A	–
	Cadario et al., 2017 [35]	A	A	–
	De Boeck et al., 2016 [42]	A	–	A
	Harkinezhad et al., 2007 [62]	E/B	E/B	–
	Heddema et al., 2006 [65]	A	A	–
	Vande Weygaerde et al., 2018 [16]	A	A	–
Strigiformes	Kalmar et al., 2014 [71]	B	B	–
Turkeys	Dickx et al., 2010 [45]	D	D	–
	Dickx et al., 2011 [46]	A, C & D	–	A & C
	Geens et al., 2005 [59]	D, F & E/B	D, F & E/B	–
	Van Droogenbroeck et al., 2009 [89]	D, E & E/B	D, E & E/B	–
	Verminnen et al., 2008 [91]	A	A	–

## Discussion

This review shows that, in addition to the traditionally reported parrot-like birds and to lesser extent pigeons, there is evidence for zoonotic transmission of *C. psittaci* from turkeys, chickens and ducks. In fact, based on our self-developed scoring system, the evidence was generally stronger for turkey and other poultry than for parrot-like birds. Moreover, zoonotic transmission from owls, peacock, geese and even mammals was reported.

Psittaciformes still remain an important source of human psittacosis, as almost one-third of the included zoonotic associations included in this review reported on psittaciformes, however, the overall strength of evidence was low. A possible explanation for our finding that the evidence for psittaciformes is relatively weak could be that clinicians and public health officials consider contact of a pneumonia patient with a parrot-like bird as sufficient evidence for psittacosis and for starting presumptive antibiotic treatment. The fact that psittacine birds are generally accepted as source of *C. psittaci* also introduces publication bias, as systematic research is performed to a lesser extent. However, when several human cases are involved and a thorough source trace-back investigation is performed, evidence for zoonotic transmission from psittaciformes can be very strong [42, 65].

In contrast, physicians might disregard turkeys and other poultry species as a source for zoonotic transmission of *C. psittaci*. ‘Natural immunity’, due to continuous exposure, has been suggested for individuals who are regularly in close contact with poultry, because in some studies most of the *C. psittaci* culture- and PCR-positive poultry workers did not present with any clinical signs [45, 46]. Contrastingly, Lagae et al. found that 25 out of 29 *C. psittaci* PCR-positive poultry farmers reported symptoms possibly related to psittacosis [73]. Since genotypes A, C and D found by Dickx et al. [46] were identical to those described by Lagae et al. [73], it is less likely that the difference between symptomatic and asymptomatic infections can be attributed to a difference in genotype. The diverging findings show that more research into asymptomatic infection and natural immunity of human psittacosis related to poultry is required.

A genotypical match between human and animal samples has been documented for chickens, columbiformes, ducks, geese, guinea fowl, passeriformes, peacock, psittaciformes, strigiformes and turkeys. Host specificity of genotypes has been described, with genotype A being mostly linked to psittacine birds, B and E to pigeons, D and E to turkeys, and C and E/B to ducks [1, 75]. Generally, these patterns of host specificity are also found in the genotypical matches summarized in this review, but exceptions are present. In turkeys, for example, genotypical matches were very divergent, as matches were also reported for the genotypes A, C and E/B, which are generally more specific for psittacine birds and ducks. Detection of a specific genotype in a human psittacosis case can give a direction for the possible animal source. However, during source tracing, also the non-genotype-host specific animal sources need to be kept in mind. Furthermore, this ‘macro’ level of genotype matching to confirm animal-human transmission has its limitations, as strain identity is less accurately defined compared to whole genome sequencing.

Results from this review also indicate the possibility of mammals being a source of *C. psittaci* infection to humans, but the strength of evidence for zoonotic transmission for these mammalian species was relatively low. The outbreak of three psittacosis cases in a veterinary school linked to exposure to infected fetal membranes of a mare did have a maximum score of 15 [37]. This could indicate a novel source of infection, but genotyping was only performed on the animal sample.

There are several reports of occurrence of *C. psittaci* in mammalian species [97–102]. However, this occurrence is often attributed to transmission from birds to mammals [100, 101, 103]. This was also suggested in the article describing zoonotic transmission from a mare, as it seemed feasible that the mare contracted the bacterium from wild birds in the surrounding area. This might indicate that mammalian species are not the reservoir of the disease, but act as an intermediate species in the transmission to humans. Nevertheless, it is important to further investigate transmission dynamics of *C. psittaci* within and between animal populations, as animal-to-animal transmission of a bovine isolate of *C. psittaci* in calves has been reported [104]. The possibility of zoonotic transmission from non-avian animals should be further investigated. Moreover, there has been evidence for human-to-human transmission of *C. psittaci* [10, 105, 106].

Recently, a *C. psittaci* related species named *C. gallinaceae* has been added to the family of *Chlamydiaceae* [107], with chickens and turkeys as the predominant hosts. Hulin et al. investigated the presence of *C. psittaci* as well as *C. gallinaceae* in poultry. They found a high prevalence of *C. gallinaceae* in a slaughterhouse where chickens, guinea fowls and turkeys were processed [68]. Human cases related to other species than *C. psittaci* were not included in this review, however, the high prevalence of *C. gallinaceae* in poultry indicates the need to assess the zoonotic potential of this relatively unknown species.

This review has some limitations. Although we included multiple languages in our search strategy, the geographical spread of articles included in our review is limited, as the majority of studies is from the European region. A relatively large number of studies originated from Belgium, the Netherlands and France, and these predominantly investigated poultry. This may reflect a particular interest in psittacosis related to poultry among researchers from these countries. Partly, this could be due to the fact that psittacosis in poultry is a notifiable disease in Belgium [108], however, not in the Netherlands and France.

We did not qualitatively review the study design of the included articles, as the majority of the included articles were case studies. This type of study design is considered of low quality and reliability. Data extraction was also difficult, as the description of human cases and animal sources was relatively poor in some studies. However, we did always extract the data according the original authors rationale.

In fifteen studies, human cases were described with multiple associated animal sources. The aim of this review was to give an overview of all associated animal sources. Therefore, in case of multiple exposures, the animal sources were entered into separate lines under the same study, which causes human cases to be entered twice. Moreover, when multiple animals are associated, it could be that for some animal species the suspicion of being the actual source of infection is low, but are included in the investigation for certainty, which can cause bias towards a lower evidence score for these animal sources.

The strength of evidence score is based on a self-developed scoring system, in which a weight was assigned to each factor included in the calculation. The subjectivity of the weights influences the strength of evidence score. With a weight of 8, the factor ‘genotypical match’ had a high impact on the final strength of evidence scores. As the genotype matching was made on a ‘macro’ level, a score of 8 is disputable. When assigning a weight of 4 to this factor, as means of a sensitivity analysis, the boxplot summary scores are lower, but the general pattern and conclusion between animal categories remain stable. For transparency, all the raw data and a flexible strength of evidence calculation tool have been included in Additional file 2. This allows the reader to manually adjust the weights and interpret the effects on the individual strength of evidence scores, as well as on the boxplot summary scores for the different animal categories.

The included studies showed a wide variety in tests used to confirm a case, e.g. PCR, serology, culture or combinations. Even between studies, that reported to use PCR, discrepancies were present, as the applied PCR methods varied in amplification techniques, specificity and DNA targets. Also the type of serological test applied (e.g. Enzyme-Linked Immunosorbent Assay, immunofluorescence, complement fixation test) differed. Apart from antigen and/or antibody testing, the specific type of test and the corresponding sensitivity or specificity values of these tests were not taken into account in our review, meaning that the reliability of the number of confirmed individuals varies per study included. We maintained the number of cases as stated in the original article, because some articles did not mention a case definition, and other articles differed too much in sampling methods and type of test to distinguish between case definitions. This broad range of tests and criteria for case confirmations is in line with the main findings of a review by Nieuwenhuizen et al. on laboratory methods for case finding in human psittacosis outbreaks [14]. They concluded that there is no standard or uniformity in tests used to confirm human cases. In general, exposure assessment in most studies was rather weak, mostly lacking specification of the chronology of events. We therefore reported ‘contact with sick animals’ irrespective of when exactly the animals became ill. People can also become infected after contact with asymptomatic animals [109], but sick animals are likely to shed more bacteria, thus having a higher chance of transmission, which is why contact with sick animals was assigned a higher weight. However, asymptomatic animals may pose a higher threat to public health because they are less evident as a source and may cause more delay in diagnosing the disease in humans.

## Conclusion

Based on our scoring system, strong evidence was found for zoonotic transmission from turkeys, chickens and ducks. The evidence was generally stronger for poultry than for parrot-like birds. One explanation could be that contact of a pneumonia patient with a parrot-like bird is often regarded as sufficient evidence, while thorough source investigation is only performed when non-traditionally reported species are implicated. Despite their low strength of evidence, psittaciformes and pigeons remain important sources of zoonotic transmission of *C. psittaci,* as is reflected by the large proportion of included studies reporting on psittaciformes and pigeons. In addition to the traditionally reported species, clinicians and public health officials should consider turkey, chicken, duck and other bird species (e.g. musophagiformes and strigiformes) as potential sources of human psittacosis cases and include these species in medical history and source tracing.

## Supplementary information

**Additional file 1.** Characteristics of 80 included studies.

**Additional file 2.** Strength of evidence tool.

## Data Availability

All data generated or analysed during this study are included in this published article and/or its supplementary information files.

## Abbreviations

C. psittaci: Chlamydia psittaci
CAP: Community acquired pneumonia
PCR: Polymerase chain reaction
